# Unexpected pulmonary tumor: metastasis from a benign uterine leiomyoma in a post-menopausal woman: a case report

**DOI:** 10.1186/s13104-017-2998-6

**Published:** 2017-12-01

**Authors:** Boubacar Efared, Gabrielle Atsame-Ebang, Rabiou Sani, Layla Tahiri, Ibrahim Sory Sidibe, Fatimazahra Erregad, Nawal Hammas, Mohamed Smahi, Mounia Serraj, Laila Chbani, Hinde El Fatemi

**Affiliations:** 1 Department of Pathology, Hassan II University Hospital, Fès, Morocco; 2 Department of Thoracic Surgery, Hassan II University Hospital, Fès, Morocco; 3 Laboratory of Translational and Biomedical Research, Faculty of Medicine and Pharmacology, Sidi Mohamed Ben Abdellah University, Fès, Morocco; 4 Faculty of Medicine and Pharmacology, Sidi Mohamed Ben Abdellah University, Fès, Morocco; 5 Department of Pneumology, Hassan II University Hospital, Fès, Morocco

**Keywords:** Benign, Metastasis, Lung, Uterus, Leiomyoma

## Abstract

**Background:**

The occurrence of lung metastasis from benign uterine leiomyomas is rarely reported especially in post menopausal women. The pathogenesis of these metastatic benign tumors still remains a subject of various speculations.

**Case presentation:**

A 57-year-old woman presented with a chronic cough and dyspnea. She had undergone 8 years previously, hysterectomy for benign leiomyomas. A chest computed tomography scan showed a 4 cm solitary nodular parenchymal tumor that increased in size after 12 months. The histological analysis of the biopsy from this nodule showed a benign tumor with regular spindle cells disposed in intersected fascicles. At immunohistochemical analysis, the tumor cells were positive for smooth muscle markers and oestrogen-progesterone receptors with a low mitotic index assessed by Ki-67. These features were consistent with a benign metastasizing uterine leiomyoma. At the multidisciplinary meeting, prescription of an aromatase inhibitor has been decided for the patient.

**Conclusions:**

Benign metastasizing uterine leiomyomas of the lung are very rare tumors. Although extremely rare in post menopausal women, their diagnosis should be considered in symptomatic patients with a history of hysterectomy for leiomyomas.

## Background

Extra-uterine locations of benign leiomyomas constitute a very rare phenomenon consisting of the occurrence of smooth muscle tumors with similar phenotype and genotype to those of benign uterine leiomyomas [1, 2]. These tumors are termed as benign metastasizing leiomyoma (BML), the term has been introduced in 1939 by Steiner [1, 3]. Since then, a few cases have been reported in the literature. Usually BML affects premenopausal women, very rarely menopausal patients, with a history of myomectomy or hysterectomy for uterine leiomyomas, and the tumors are discovered incidentally during a routine follow-up or rarely diagnosed after clinical symptoms [2, 4–6]. Also, BML can be diagnosed concomitantly with a uterine leiomyoma [1, 2, 7]. Lungs are the most frequent site of secondary locations of BML, but other sites can be involved such as peritoneum, retroperitoneum, cardiovascular system, spine, or soft tissues [1, 8–11]. The period between the diagnosis of uterine leiomyomas and the discovery of BML varies widely as these tumors can be diagnosed at the same time or BML can occur many years later [1–3, 11]. The pathogenesis of BML still remains a subject of controversies and speculations. In fact, it is well accepted that BML are tumors of smooth muscle origin with similar histological, immunohistochemical and molecular patterns, to those of benign uterine leiomyomas [2, 3, 12]. The enigmatic questions are the mechanisms of extra-uterine locations of this benign smooth muscle tumor. Coelomic metaplasia has been postulated to explain certain locations like peritoneal, cardiac or pleural BML; hormonal stimulation of local smooth muscle, metastasis of a low grade uterine leiomyosarcoma or transportation of leiomyoma’s cells through lymphovascular system, have been postulated as a spread mechanism of the tumors [1–3, 13]. The transportation theory seems to be more appropriate as lungs and cardiovascular system are the most affected organs by BML [2, 14].

Actually, there are no specific guidelines for BML treatment. Surgery for therapeutic and diagnostic purposes has been widely reported. Hormonal manipulation (oophorectomy or drug prescriptions) is also another therapeutic option [1, 13].

We present herein, a case of a BML of the lung in a 57-year-old symptomatic postmenopausal woman, previously treated by hysterectomy for a uterine leiomyomatous tumor.

## Case presentation

**Fig. 1:**
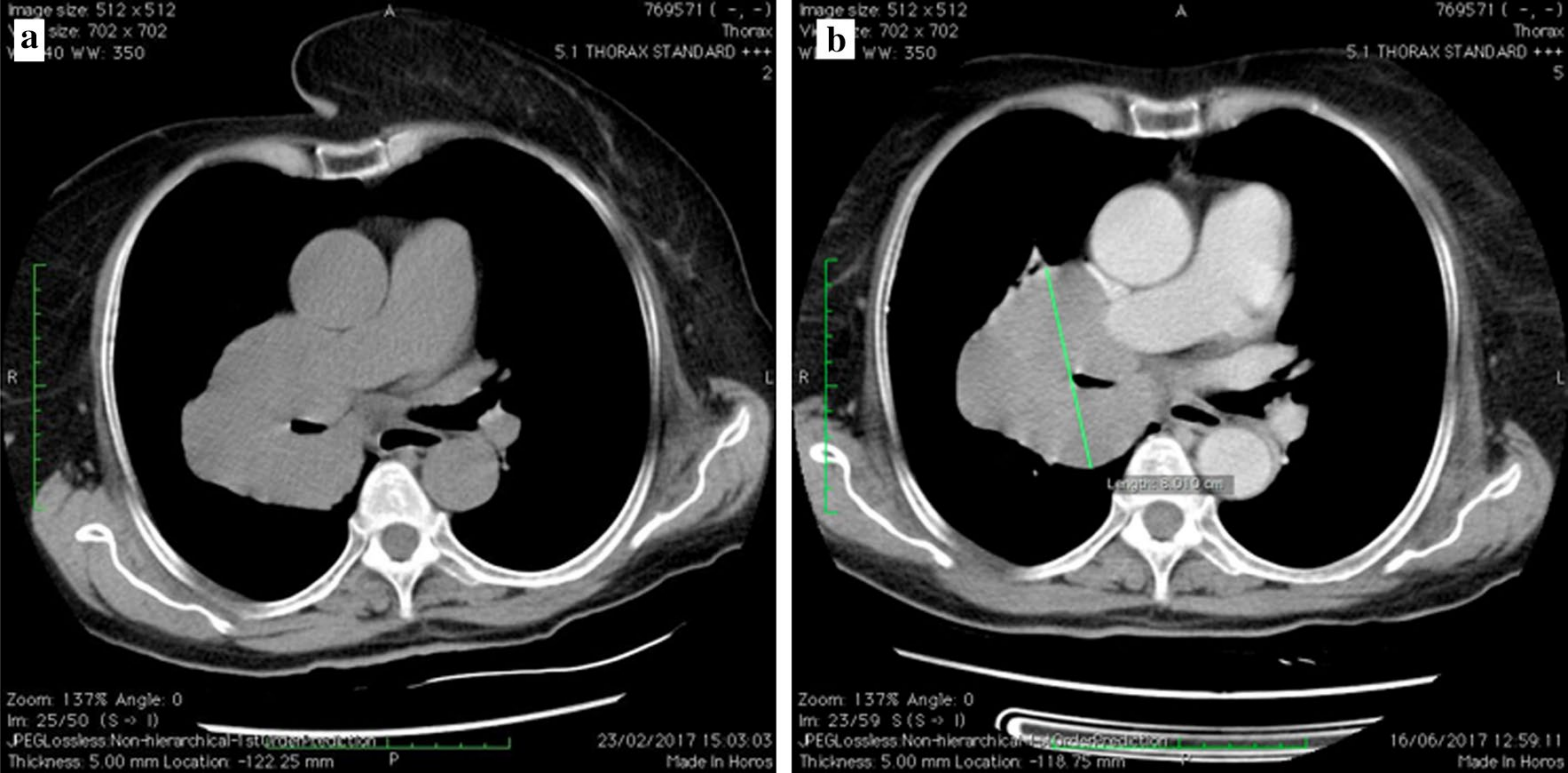
CT-scan after 1 month and half of treatment showed a well demarcated and homogeneous right proximal tumor with a bronchial compression. The tumor measures about 8 cm (**a**). After 5 months of treatment, the tumor presents the same features (**b**)

A 57-year-old woman presented with a chronic cough and dyspnea. The patient had a history of a total hysterectomy for leiomyoma 8 years ago. The histological diagnosis was benign leiomyoma. A thoracic computed-tomography scan (CT-scan) showed a 4 cm solitary, intraparenchymal and proximal nodular tumor of the right lung. The histological analysis of biopsies from this nodule revealed a benign leiomyoma of the lung. At multidisciplinary meeting (MDM), a regular surveillance has been decided as the surgery would be a total pneumonectomy given the proximal location of the tumor. The patient has been lost to follow-up as she did not come back to hospital for her regular surveillance. However, a year later, she was back to hospital for increased cough, dyspnea and a chest pain. The physical examination revealed a shortness of breath with wheezing at respiratory auscultation of the right side of the thoracic wall. There was no other organomegaly detected. A new CT-scan showed a significant increase in size (of about 4 cm) of the initial tumor discovered a year ago, with compression of the right bronchus (Fig. 1). A leiomyosarcomatous transformation has been suspected and biopsies have been performed. The histopathological analysis disclosed the diagnosis of a benign metastasizing leiomyoma (BML) of the lung. The prescription of an anti-aromatase drug has been decided at the MDM for the patient (letrozole 2.5 mg, 1 tablet per day). Six months after this treatment, the patient had no clinical symptoms and the tumor had stable size (8 cm) on CT-scan (Fig. 1b).

### Histopathological analysis

The histological analysis has been performed on a paraffin-embedded and formalin fixed tissue stained by hematoxylin–eosin-saffron (HES). The immunohistochemical analysis has also been performed on paraffin-embedded and formalin fixed tissue. We have used the antibodies according to the manufacturer’s guidelines, with an automated immunohistochemical stainer (Ventana BenchMark ULTRA^®^). At our pathology department, for all antibodies, positive and negative controls were routinely performed, including processing of normal tissue or tumor sections known to be positive (normal breast tissue, breast carcinomas, normal uterine wall, normal nerve tissue, etc.). We have used these antibodies: anti-smooth muscle actin (1A4, Cell Marque^®^), anti-desmin (DE-R-11, Ventana^®^), anti-Ki-67 (30-9, Ventana^®^), anti-Estrogen receptor (SP1, Ventana^®^), anti-Progesterone receptor (1E2, Ventana^®^), anti-CD34 (QBEnd/10, Ventana^®^), anti-S100P (16/f5, Cell Marque^®^).

**Fig. 2:**
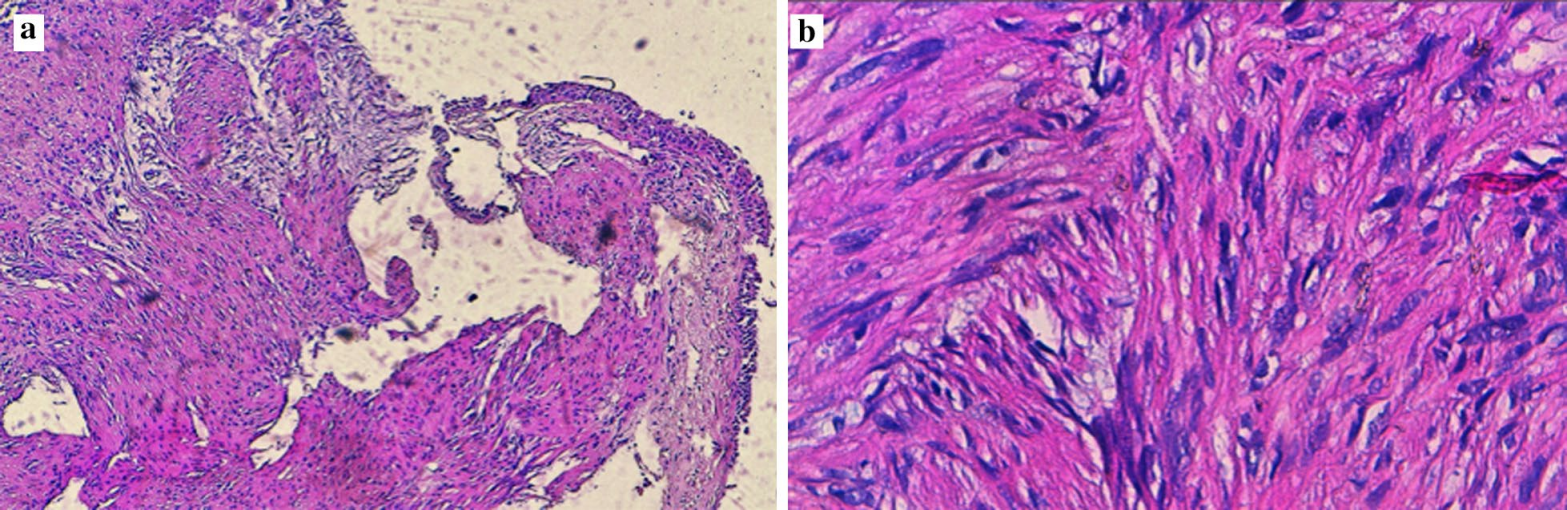
The histological images show a benign proliferation made of spindle cells disposed in intersected fascicles beneath a respiratory mucosa (**a**, hematoxylin–eosin saffron ×100). Tumor cells have elongated cigar-like nucleis with minimal atypia and without mitotic figures (**b**, hematoxylin–eosin saffron ×400)

The histological examination showed a similar pattern with the initial biopsies submitted previously. It consisted of a benign proliferation made of spindle cells disposed in intersected fascicles beneath the respiratory mucosa. Tumor cells had elongated cigar-like nucleis with minimal atypia and without mitotic figures. Tumor cells had amphophilic to eosinophilic cytoplasm with ill-defined borders (Fig. 2a, b). There was no tumor necrosis.

**Fig. 3:**
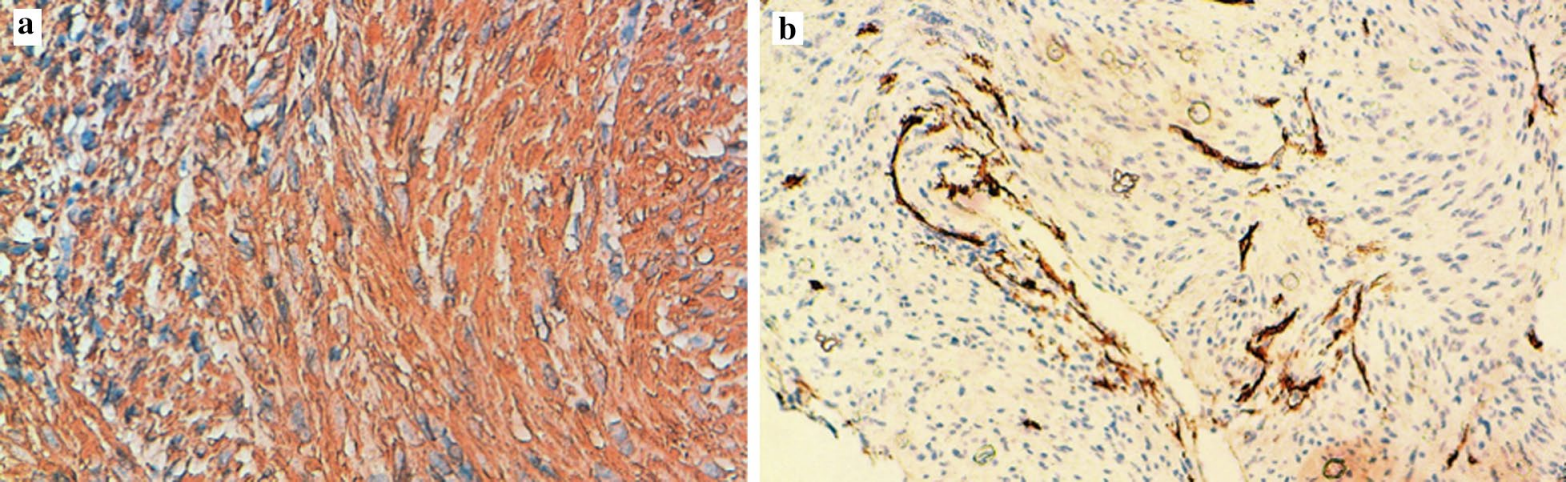
Tumor cells stain positive for SMA (**a**) (immunohistochemistry ×400), and negative for CD34 that highlights some vessels of the tumor stroma (**b**) (immunohistochemistry ×100)

**Fig. 4:**
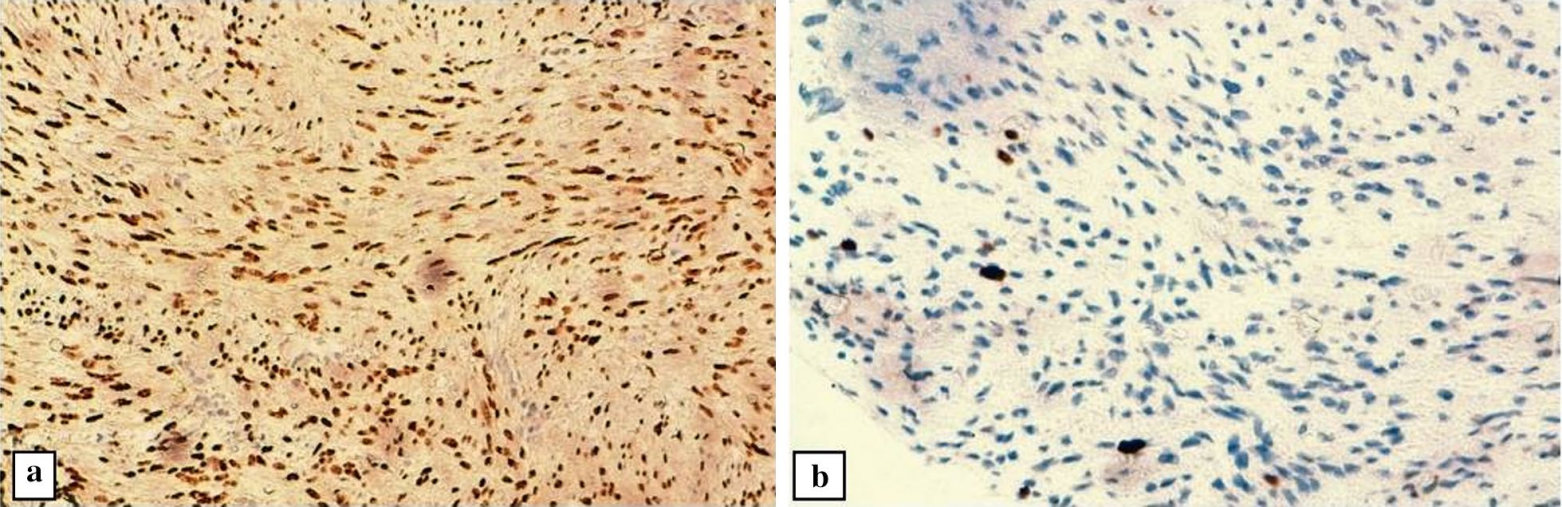
At immunohistochemistry, tumor cells are strongly positive for oestrogen receptor (**a**), with a low mitotic rate as assessed by Ki-67 (**b**), (× 200)

At immunohistochemistry, tumor cells showed a diffuse and strong staining for desmin, smooth muscle actin (SMA) (Fig. 3a), oestrogen receptor (Fig. 4a) and progesterone receptor. Tumor cells were negative for CD34 (Fig. 3b) and S-100 protein. The Ki-67 index was low, approximately 5% (Fig. 4b). Based on these histopathological patterns, the diagnosis of a benign metastasizing leiomyoma (BML) of the lung has been made.

## Discussion and conclusions

Benign metastasizing leiomyomas are tumors supposed to derive from uterine leiomyomas, that secondarily metastasize to distant organs. Uterine leiomyomas are smooth muscle derived benign tumors that are mostly encountered in women with reproductive age [3, 13, 14]. Rare cases of BML in post menopausal women have been reported, like our case, implying that other factors beyond hormones could play a major role in the pathogenesis of these tumors [6]. Benign leiomyomas are the main cause of hysterectomy or myomectomy. Secondary locations occur years after the initial surgical treatment or curetage, varying approximately from 3 to 36 years in the literature [1–3]. Usually, BML are diagnosed in premenopausal women, however rare cases of BML have been reported from post-menopausal women, like our current case of a 57-year-old woman who underwent hysterectomy for benign leiomyomas 8 years ago.

Pulmonary BML are often diagnosed incidentally by imaging techniques, rarely patients present with clinical symptoms. Respiratory symptoms like shortness of breath, cough or chest pain are very rarely reported [1]. The radiological aspects of the BML, especially on x-rays or computed-tomography scan (CT-scan), consist of solitary or multiple small lung nodules that are well circumscribed, without calcifications or contrast enhancement on CT-scan. Their size ranges often from millimeters to centimeters and frequently patients present with bilateral lung nodules [3, 8]. These features could be easily mistaken for any secondary malignant tumor of the lung, especially when the patient has a history of a malignant tumor elsewhere, or a concomitant tumor. Recently, Kim JJ et al. have reported a case of a 52-year-old woman that had undergone hysterectomy and bilateral salpingo-oophorectomy for ovarian endometrial adenocarcinoma. The uterine showed some benign leiomyomas. However, the patients had also lung nodules that have been considered to be metastasis of the patient’s ovarian carcinoma. Resection of these lung nodules has been performed and, surprisingly, the histopathological analysis has shown that these nodules were metastasis from the patient’s uterine leiomyomas [7]. In fact, in our case, the patient had a solitary pulmonary nodule with clinical symptoms that worsened over the time, suggesting a primary lung tumor. Although more frequent, the lungs are not the exclusive localisations of BML. Other rare sites have been described, such as lymph nodes, the spine, the retroperitoneum, the heart, bones or the skin [1, 4, 8–11].

As radiological features are not specific, the histopathological examination remains the unique way to confirm the diagnosis of the BML. The pathological diagnosis can be achieved by biopsy or surgical specimens of the tumor. In fact, BML is not a malignant transformation of the uterine leiomyomas. These tumors have the same histologic characteristics as the initial uterine neoplasms and present as a well differentiated smooth muscle tumor with spindle cells disposed in intersected fascicles or in a diffuse pattern. Tumor cells have a benign appearance without significant nuclear atypia, mitosis or necrosis [2]. However, these morphological features are not specific, as many mesenchymal tumors, or even epithelial tumors, can present with similar aspects. Immunohistochemical analysis is required for an accurate diagnosis. The typical immunophenotype of BML is expression of smooth muscle markers, oestrogen and progesterone receptors, as well as a very low mitotic index assessed by Ki-67. The usually used smooth muscle markers are desmin, alpha-smooth muscle actin (SMA) or H-caldesmone. Also tumor cells can be positive for S-100 protein, CD10, or bcl-2 [2, 3, 12]. The main histological differential diagnosis are other smooth muscle tumors such as metastatic leimyosarcoma of the uterine that can have a similar immunophenotype, but with usually prominent cellular atypia, necrosis and a high mitotic index. Other extra-uterine smooth muscle tumors lack oestrogen-progesterone receptors expression. A solitary lung fibrous tumor can have morphologic patterns similar to those of BML, but tumor cells are often positive for CD34 and negative for oestrogen receptors and smooth muscle markers. Other differential diagnosis include inflammatory myofibroblastic tumors, monophasic synovial sarcoma, metastatic gastrointestinal stromal tumors (GIST), …etc. [1–3]. However, a minimal immunohistochemical assessment can easily achieve a correct diagnosis by using smooth muscle markers and oestrogen-progesterone receptors. Our current case has immunohistochemical features consistent with BML, as tumor cells were positive for smooth muscle markers and hormonal receptors, and negative for CD34. But, the initial biopsy of our patient failed to show positive immunostaining for hormonal receptors, leading to the diagnosis of the lung leiomyoma. This mistake was perhaps due to errors in immunohistochemical techniques.

The pathophysiology of BML is not well elucidated an still to be a subject of many speculations. The most reported theory is the “transportation theory” [14]. According to this theory, tumor cells reach distant organs by vascular or lymphatic vessels, usually after iatrogenic manipulations (surgery or curetage). Another postulated theory, is that BML constitutes in fact metastasis from underdiagnosed low-grade uterine leiomyosarcomas. However BML are indolent and slow-growing tumors and have not histological and biological features of leiomyosarcomas, contradicting the theory of metastatic leiomyosarcomas. Coelomic metaplasia by transformation of the sub-epithelial fibrous tissue in smooth muscle tissue, under hormonal influence, has been postulated to explain BML occurence in mesothelial tissues like the pleura, the pericardium and the peritoneum. Another theory, “peritoneal seeding”, likely valid for peritoneal BML, is implantation of tumor cells in the peritoneal serosa after surgical hysterectomy, especially by the morcellation procedure, or even after a ruptured gravidic uterus [2].

The management of the BML is not well defined. As indolent tumors with a low progression rate, a long radiological surveillance is required, as tumors can regress after the menopause. Symptomatic cases should be treated by surgical lung resection or by hormonal manipulation (oophorectomy or drugs like aromatase inhibitors, gonadotropin-releasing hormone agonists, …etc.) [2, 13]. Cases that have progressed under hormonal manipulation have been reported, especially in post menopausal women [4]. This fact highlights the unresolved issue of the pathogenesis of BML, and further studies in the future are needed to uncover the mystery of these benign metastatic neoplasms.

In summary, metastasizing uterine leiomyomas of the lung are very rare neoplasms with clinicopathological features of benign tumors. Although extremely rare in post menopausal women, their diagnosis should be considered in any patient with pulmonary symptoms and a history of hysterectomy for leiomyomatous tumor.

## Abbreviations

BML: benign metastasizing leiomyoma
GIST: gastrointestinal stromal tumors
MDM: multidisciplinary meeting
SMA: alpha-smooth muscle actine
